# LPS Remodeling Triggers Formation of Outer Membrane Vesicles in *Salmonella*

**DOI:** 10.1128/mBio.00940-16

**Published:** 2016-07-12

**Authors:** Wael Elhenawy, Michael Bording-Jorgensen, Ezequiel Valguarnera, M. Florencia Haurat, Eytan Wine, Mario F. Feldman

**Affiliations:** a Department of Biological Sciences, University of Alberta, Edmonton, Alberta, Canada; b Department of Physiology, University of Alberta, Edmonton, Alberta, Canada; c Department of Molecular Microbiology, Washington University School of Medicine, St. Louis, St. Louis, Missouri, USA; d Department of Pediatrics, University of Alberta, Edmonton, Alberta, Canada

## Abstract

Outer membrane vesicles (OMV) are proposed to mediate multiple functions during pathogenesis and symbiosis. However, the mechanisms responsible for OMV formation remain poorly understood. It has been shown in eukaryotic membranes that lipids with an inverted-cone shape favor the formation of positive membrane curvatures. Based on these studies, we formulated the hypothesis that lipid A deacylation might impose shape modifications that result in the curvature of the outer membrane (OM) and subsequent OMV formation. We tested the effect of lipid A remodeling on OMV biogenesis employing *Salmonella enterica* serovar Typhimurium as a model organism. Expression of the lipid A deacylase PagL resulted in increased vesiculation, without inducing an envelope stress response. Mass spectrometry analysis revealed profound differences in the patterns of lipid A in OM and OMV, with accumulation of deacylated lipid A forms exclusively in OMV. OMV biogenesis by intracellular bacteria upon macrophage infection was drastically reduced in a *pagL* mutant strain. We propose a novel mechanism for OMV biogenesis requiring lipid A deacylation in the context of a multifactorial process that involves the orchestrated remodeling of the outer membrane.

## IMPORTANCE

The role of lipid remodeling in vesiculation is well documented in eukaryotes. Similarly, bacteria produce membrane-derived vesicles; however, the molecular mechanisms underlying their production are yet to be determined. In this work, we investigated the role of outer membrane remodeling in OMV biogenesis in *S*. Typhimurium. We showed that the expression of the lipid A deacylase PagL results in overvesiculation with deacylated lipid A accumulation exclusively in OMV. An *S*. Typhimurium Δ*pagL* strain showed a significant reduction in intracellular OMV secretion relative to the wild-type strain. Our results suggest a novel mechanism for OMV biogenesis that involves outer membrane remodeling through lipid A modification. Understanding how OMV are produced by bacteria is important to advance our understanding of the host-pathogen interactions.

## INTRODUCTION

Outer membrane vesicles (OMV) are liposomal structures protruding from the outer membrane (OM) of Gram-negative bacteria. They have been proposed to mediate numerous functions in environmental bacteria, pathogens, and symbionts (1, 2). These roles involve quorum sensing, horizontal gene transfer, interbacterial killing, toxin delivery, hydrolysis of polysaccharides, and secretion of misfolded proteins to relieve cell envelope stress (1–7). Several models for OMV biogenesis have been suggested (1, 2, 8, 9). However, the molecular mechanisms leading to OMV formation are not well understood. OMV share the basic components with the parent OM, including OM proteins, phospholipids, and lipopolysaccharides (LPS). Despite the similarity in general composition between the OM and OMV, OMV appear to carry specific protein cargo in some bacteria (1, 10–15). Moreover, lipid analysis of the two compartments in several bacteria suggested the enrichment of specific lipid species in OMV compared to the OM (9, 13, 16, 17). These observations imply that OM remodeling occurs before the blebbing of the OMV. We have shown that OMV produced by the dental pathogen *Porphyromonas gingivalis* are enriched in deacylated lipid A forms compared to the lipid A purified from the cells (14). Lipid A is an acylated disaccharide and constitutes the hydrophobic anchor of LPS in the outer leaflet of the OM. Because lipid A deacylation is best studied in *Salmonella* (18–20), we chose this organism to test our model that lipid A deacylation is involved in OMV formation.

*Salmonella enterica* serovar Typhimurium (referred to as *S*. Typhimurium) is a leading cause of gastroenteritis, with approximately 3 million deaths reported annually worldwide (21). *S*. Typhimurium possesses a plethora of virulence factors that are required for disease development. Many of these virulence factors are encoded within horizontally acquired genomic islands called *Salmonella* pathogenicity islands (SPI) (22). SPI-1 and SPI-2 are the main virulence determinants. Each of them encodes a different type III secretion system (T3SS), which is a type of nanomachinery capable of injecting bacterial effectors across the eukaryotic plasma membrane into the host cell cytosol (23–27). In addition to T3SS, it has been proposed that intracellular *S*. Typhimurium can secrete its proteins via OMV (28). To minimize fitness trade-offs *in vivo*, *S*. Typhimurium relies on two-component regulatory systems to spatially and temporally regulate the expression of genes within different SPIs (29). Following phagocytosis by macrophages, *S*. Typhimurium resides in a specialized compartment, termed the *Salmonella*-containing vacuole (SCV). The intravacuolar milieu activates the two-component system, PhoPQ (30). Once activated, PhoPQ mediates lipid A modifications and OM remodeling (31–33).

The secretion of LPS-positive vesicles by intracellular *S*. Typhimurium has been reported before (28, 34). A previous study demonstrated the release of LPS by *S*. Typhimurium into the infected epithelial cells. The secreted LPS was detected in multiple cellular compartments, including the SCV and the host cell cytosol (34). Recently, Guidi et al showed that *S*. Typhimurium can secrete OMV packed with cytolethal distending toxin (CDT) inside infected epithelial cells. The secreted vesicles tested positive for *S*. Typhimurium LPS (28). Together, these results suggested that *S*. Typhimurium produces OMV during its intracellular life.

Employing biophysical methods, it has been established that fully acylated and deacylated lipid A adopt different spatial configurations due to the variation in the cross sections of their hydrophobic moieties. Hexa-acylated lipid A is conical in shape, while the deacylated forms tend to acquire cylindrical to inverted-cone-shaped structures (35). The influence of lipid geometric properties on membrane curvature is well studied in eukaryotes (36). Lipids with inverted-cone-shaped structures, like lysophosphatidylcholine, favor the formation of positive membrane curvatures (37). Based on these studies, we formulated the hypothesis that lipid A deacylation might impose shape modifications that result in the curvature of the OM and subsequent OMV formation. The *S*. Typhimurium enzyme PagL removes the β-hydroxymyristoyl chain in the 3 position of lipid A and is tightly regulated by the PhoPQ two-component system (19). This modification makes lipid A, and hence *S*. Typhimurium, less detectable by Toll-like receptor 4 (TLR-4) of the host immune cells (18). Activation of PhoP-PhoQ also activates a second two-component regulatory system, PmrA-PmrB, which promotes the attachment of aminoarabinose and phosphoethanolamine to the phosphate groups on lipid A. PagL-modified lipid A is not detected in PhoPQ-activating conditions *in vitro*, but can occur in PmrA or PmrB mutants, both of which lack the aminoarabinose modification of lipid A. Because of the inhibitory effect of the aminoarabinose lipid A modifications on PagL, the enzyme is considered to be “latent” (38, 39).

**FIG 1:**
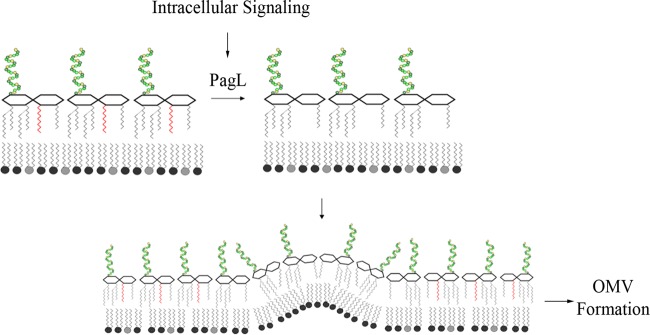
Hypothetical model for PagL-mediated vesiculation in *S*. Typhimurium. During its intracellular life, *S*. Typhimurium will activate PagL in response to host cues. PagL will deacylate lipid A, leading to a change in its topology. As a consequence, with the decrease in hydrophobic cross-section area, lipid A will adopt an inverted-cone shape, leading to membrane curvature and OMV formation.

In this work, we tested the hypothesis that accumulation of deacylated lipid A molecules in the OM produced by PagL can induce OMV formation (Fig. 1). We tested our model *in vitro* using *S*. Typhimurium expressing active and inactive variants of PagL. Moreover, we investigated the role of PagL in intracellular OMV production.

## RESULTS

### OM and OMV share the same lipid A profile in the absence of PagL expression.

We initially compared the profiles of the lipid A extracted from bacterial cells harvested from *S*. Typhimurium grown *in vitro* under standard lab conditions with the profile of the lipid A molecules obtained from OMV by mass spectrometry (MS). This analysis revealed identical profiles in both the cellular and OMV fractions. As shown in Fig. S1 in the supplemental material, the *bis*-phosphorylated hexa-acylated lipid A was the predominant peak (*m*/*z* 1,796) in both spectra. In agreement with previous reports, we did not detect any PagL-mediated deacylated forms of lipid A in *S*. Typhimurium cells and OMV, which was expected as PagL is not induced under the conditions employed (38).

### Construction of catalytically inactive variant of PagL.

To study the effect of PagL on vesiculation *in vitro*, we adopted a biochemical approach. Instead of growing the bacteria under PhoPQ-activating conditions, we expressed PagL recombinantly in *S*. Typhimurium. This approach enabled us to study the effect of PagL on OMV formation in the absence of other PhoPQ-mediated lipid A modifications (40, 41).

To investigate whether lipid A deacylation can lead to OMV formation, we cloned and expressed a His-tagged version of PagL. To distinguish between possible effects caused by PagL expression and not by its activity, we created a catalytically inactive variant of PagL. Geurtsen et al. identified the conserved residues among PagL homologues in different bacteria (42). Another study revealed the catalytic mechanism for PagL and demonstrated that histidine (position 163) and serine (position 165) residues are essential for activity (43). Therefore, we performed site-directed mutagenesis to change the latter residues to alanine, which should abolish the deacylation activity. To confirm that PagL_H163A S165A_ was enzymatically inactive, we employed a modified *in vitro* assay for lipid A deacylation. We expressed PagL and PagL_H163A S165A_ in *Escherichia coli* DH5ɑ, which lacks a homologue for *pagL* in the genome (19, 42). Membrane preparations of different *E. coli* strains expressing PagL, PagL_H163A S165A_, and the empty vector were incubated with purified *S*. Typhimurium LPS overnight. Following incubation, lipid A was extracted from the different reactions and analyzed by MS. Only the expression of wild-type PagL resulted in the detection of a peak corresponding to the 3-*O*-deacylated *bis*-phosphorylated lipid A (*m*/*z* 1,570). Conversely, the mutated variant of PagL_H163A S165A_ (now referred to as PagL_inactive_) lost its ability to deacylate lipid A, as indicated by the absence of the *m*/*z* 1,570 peak (see Fig. S2 in the supplemental material).

### Overexpression of active PagL increases OMV production.

**FIG 2:**
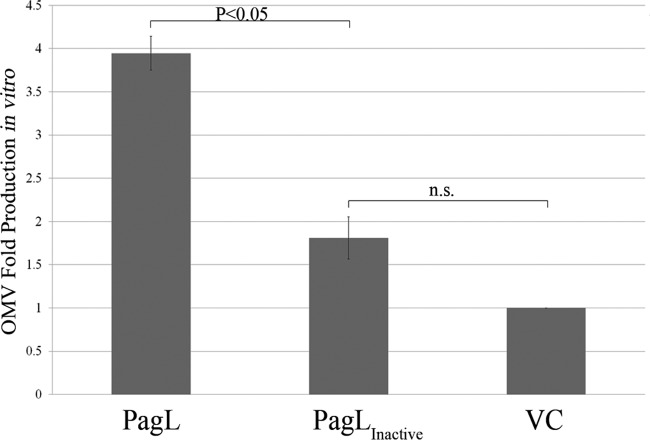
*In vitro* expression of catalytically active PagL induces OMV production in *S*. Typhimurium. Shown is OMV fold production by *S*. Typhimurium strains expressing PagL or PagL_inactive_ relative to the control strain carrying the empty vector (VC). OMV preparations were obtained from OD_600_-normalized cultures, and KDO content was determined using a colorimetric assay. The results were obtained from three independent experiments under the same conditions. Presented are means ± standard errors of the means (SEM [*n* = 3]). Statistical significance was determined using ANOVA followed by Tukey’s test.

**FIG 3:**
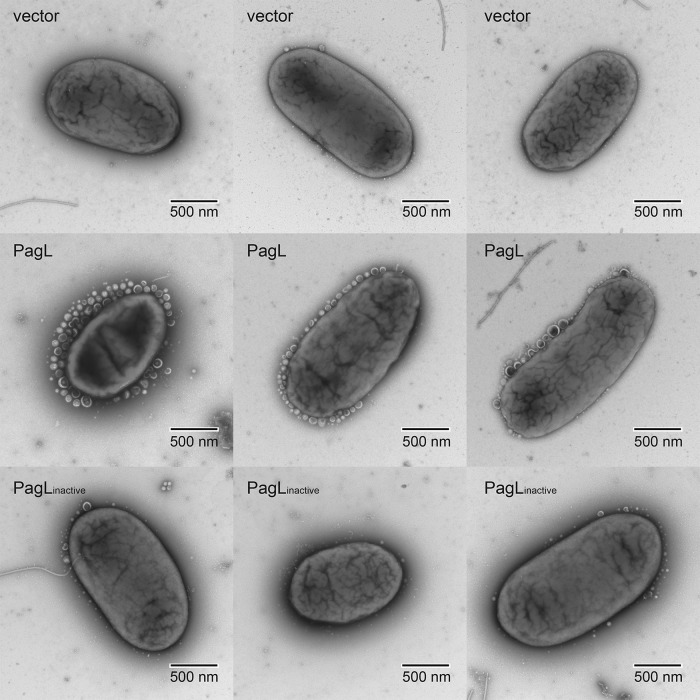
PagL expression induces OMV budding. *S*. Typhimurium cells grown in LB agar plates with 0.5 mM IPTG were resuspended in PBS and visualized by transmission electron microscopy. vector, pEXT21 empty vector control; PagL, vector expressing active form of PagL; PagL_inactive_, vector expressing inactive variant of PagL. Panels are representative of 40 images acquired per strain using a ×15,000 magnification.

To examine if lipid A deacylation is related to vesiculation, we measured OMV production by *S*. Typhimurium transformants expressing PagL and PagL_inactive_ for 4 h from a low-copy-number vector. Using immunoblotting, we verified that both PagL and PagL_inactive_ were expressed at equivalent levels (see Fig. S3 in the supplemental material). Moreover, OMV were harvested from cell-free supernatants of cultures normalized to the optical density at 600 nm (OD_600_). Other studies relied on measuring the protein concentration of OMV preparations as a representative of vesiculation levels (6, 44–46). However, this approach does not take into account that proteins, such as flagellins, associate in a nonspecific manner with OMV, which can mask the results. Alternatively, we employed a specific colorimetric assay to quantify the 3-deoxy-d-manno-octulosonic acid (KDO) content in the OMV (47). KDO is bound to lipid A at position 6′ in many bacterial species (48). In *S*. Typhimurium, two molecules of KDO covalently modify each molecule of lipid A (49). Since lipid A is a principal component of OMV, KDO levels are representative of the amounts of OMV produced. As shown in Fig. 2, PagL expression resulted in about a 4-fold increase in OMV production compared to that of the vector control. The expression of PagL_inactive_ did not significantly increase OMV production, compared to the vector control (Fig. 2). To visualize vesiculation *in vitro*, we employed transmission electron microscopy (TEM). Strains carrying PagL and its inactive variant were grown overnight on solid media. Bacteria were directly resuspended in phosphate-buffered saline (PBS) and fixated for TEM. Figure 3 shows bacteria representative of multiple acquired images. Expression of PagL resulted in the presence of abundant OMV surrounding the cells. These OMV were remarkably homogeneous in size (approximately 50 to 120 nm). Fewer OMV were seen in cells expressing the inactive variant of PagL or the vector alone, in agreement with the amounts of KDO levels detected in the supernatants. Together, these experiments suggest that PagL activity leads to OMV formation in *Salmonella* cells.

To examine if the increase in OMV production upon PagL overexpression is the result of envelope stress due to membrane instability, we monitored three of the main systems involved in the bacterial envelope stress response: the σ^E^, Cpx, and Rcs pathways (50, 51). We used quantitative real-time PCR (RT-qPCR) to measure the effect of expressing PagL on the transcriptional levels of key components in each pathway: *rpoE*, *cpxR*, and *rcsC*, which are upregulated in response to the activation of their corresponding pathways (50–52). *rpoE* codes for the main transcription factor required for the upregulation of the σ^E^ regulon during envelope stress. CpxR is the response regulator in the Cpx pathway, while RcsC is a key histidine kinase in the Rcs phosphorelay system. None of the previous genes was upregulated when PagL, or its inactive variant, was overexpressed, indicating that PagL expression does not induce any of the envelope stress responses known in *S*. Typhimurium (see Fig. S4 in the supplemental material).

### PagL expression leads to accumulation of deacyated lipid A in OMV.

**FIG 4:**
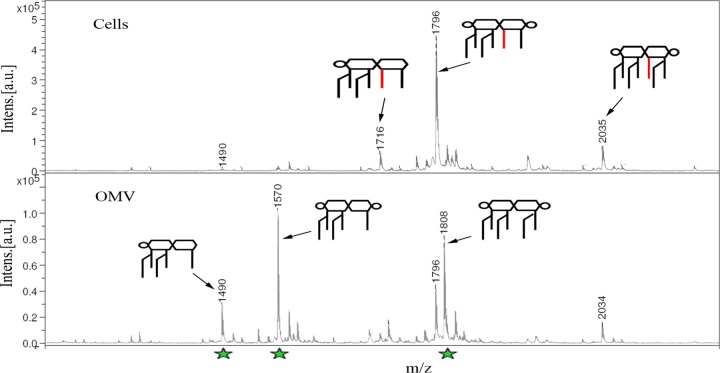
Deacylated lipid A is preferentially packed into *S*. Typhimurium OMV when PagL is expressed. Lipid A was purified from cells and OMV of an *S*. Typhimurium strain expressing PagL from pWel2. MS analysis of the purified lipid A revealed the accumulation of deacylated lipid A species (marked by green stars) in OMV compared to cells. The major deacylated lipid A species detected in OMV are *bis-*phosphorylated hexa-acylated lipid A (*m*/*z* 1,808), *bis-*phosphorylated penta-acylated lipid A (*m*/*z* 1,570), and monophosphorylated penta-acylated lipid A (*m*/*z* 1,490). On contrary, the major lipid A forms that were detected in cells had the β-hydroxymyristoyl group at the 3 position of lipid A. The main cellular lipid A species detected were *bis-*phosphorylated hepta-acylated lipid A (*m*/*z* 2,035), *bis-*phosphorylated hexa-acylated lipid A (*m*/*z* 1,796), and monophosphorylated hexa-acylated lipid A (*m*/*z* 1,716). Intens., intensity; a.u., arbitrary units. These results are representative of two independent experiments.

The previous results suggested a possible role of PagL in OMV formation. According to our model, localized lipid A deacylation at certain regions of the OM would cause its curvature. Consequently, the released vesicles should be enriched in deacylated lipid A. We compared the lipid A contents of OM and OMV of *S*. Typhimurium upon overexpression of PagL. Lipid A was purified and subjected to MS analysis. In the control strain carrying the empty vector, both fractions displayed the same lipid A species, and the spectra were similar to the ones obtained for cells that did not carry the plasmid (see Fig. S5 in the supplemental material). However, expression of PagL resulted in striking differences between the cellular lipid A and that purified from secreted OMV. *bis*-Phosphorylated hexa-acylated lipid A (*m*/*z* 1,796) was the predominant lipid A variant in the cellular fraction of PagL-expressing bacteria (Fig. 4). This was also the main lipid A form seen in bacteria not expressing PagL (see Fig. S1 in the supplemental material). Other less predominant forms were detected in the cells, including the mono-phosphorylated hexa-acylated (*m*/*z* 1,716) and the *bis*-phosphorylated hepta-acylated lipid A (*m*/*z* 2,035). The formation of the hepta-acylated lipid A is mediated by PagP, and an acyltransferase that attaches a palmitoyl group from a phosphatidylglycerol donor to the 2 position in lipid A (53). Importantly, the major peaks detected in the OMV fraction corresponded to deacylated forms of the lipid A species present in the OM. The *bis*-phosphorylated penta-acylated lipid A (*m*/*z* 1,570) was the most predominant form in OMV and corresponds to the *m*/*z* 1,796 form minus the β-hydroxymyristoyl substituent acyl chain removed by PagL. Furthermore, the mono-phosphorylated penta-acylated lipid A (*m*/*z* 1,490) and the *bis*-phosphorylated hexa-acylated lipid A (*m*/*z* 1,808) were also prominent in the OMV. These lipid A modifications were all PagL dependent since we did not detect these forms in the *S*. Typhimurium strain carrying the empty vector.

### PagL is required for OMV formation by intracellular *S*. Typhimurium.

**FIG 5:**
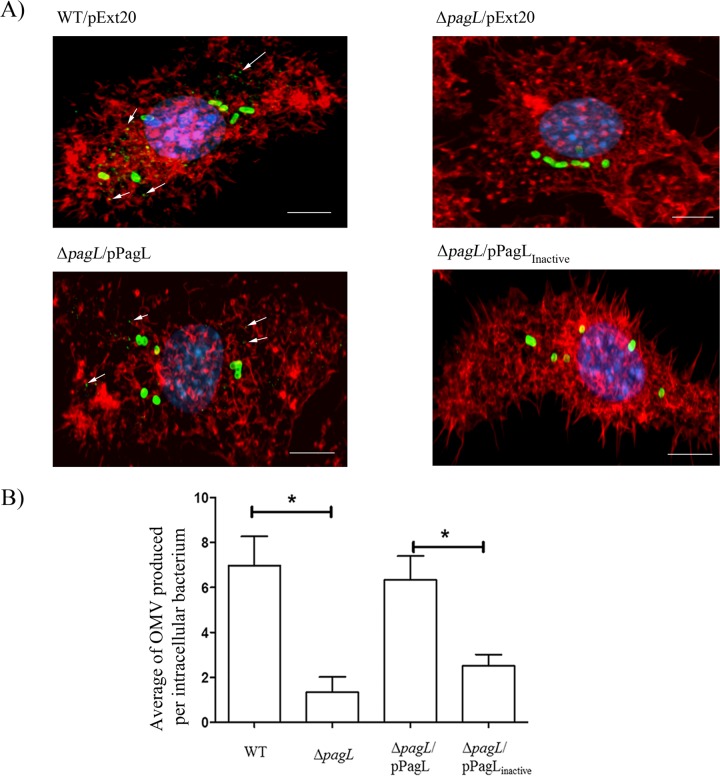
PagL plays a role in intracellular vesiculation of *S*. Typhimurium. Shown are results from immunofluorescence-based detection of OMV (marked by white arrows) production in J774A.1 mouse macrophages infected with different *S*. Typhimurium strains. Presented are the combined z-stacks for each infected cell. After incubation, cells were fixed with 4% PFA, stained with monoclonal antiabequose to detect *S*. Typhimurium LPS (green), actin-specific phalloidin (red), and DAPI (blue). Each image shown represents a set of 30 images. Scale bars represent 5 µm (A). Intracellular OMV of different strains were counted using ImageJ and normalized by the number of OMV-producing bacteria in each macrophage. Presented are the mean values obtained from 30 images (B). Statistical significance (marked by asterisks) was determined using ANOVA followed by Tukey’s test (*P* < 0.05).

**FIG 6:**
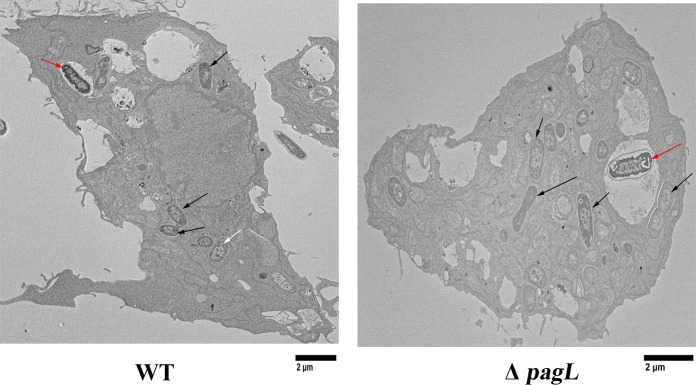
Deletion of *pagL* did not alter the subcellular localization of *S*. Typhimurium. Shown is TEM examination of J774A.1 mouse macrophages at 4 h postinfection with different *S*. Typhimurium strains. The majority of intracellular bacteria were detected in the cytoplasm (marked by black arrows). Examples of intravacuolar bacteria are marked with red arrows. Each panel is representative of 15 different images obtained from 2 biological replicates.

Our biochemical analysis strongly supported the hypothesis that PagL-mediated remodeling of LPS increases vesiculation. To investigate the effect of PagL activity on vesiculation under physiological conditions, we compared the wild type and its isogenic Δ*pagL* mutant strain for OMV production in J774A.1 mouse macrophages. Employing a monoclonal antibody highly specific for the abequose residue present in *S*. Typhimurium O antigen, we visualized the intracellular LPS by immunofluorescence. Following the staining of actin cytoskeleton in infected macrophages, we employed confocal microscopy to detect intracellular bacteria and OMV. In agreement with previous reports, our microscopy analysis revealed the presence of material, compatible in size with OMV, that tested positive for LPS inside the infected macrophages. These vesicles were detected in close vicinity to and also apart from the intracellular bacteria (Fig. 5A). We quantified the levels of intracellular OMV production by the two strains employing the ImageJ software to count LPS-positive vesicles of diameter 250 nm and below (54). We chose the diameter of 250 nm as a cutoff for OMV size in agreement with previous reports (2). We normalized the counted OMV by the number of intracellular bacteria within the same macrophage. A total of 30 images per strain were used to measure the average amounts of OMV produced per intracellular bacterium. Importantly, any macrophages with LPS-positive particles between 250 nm and 0.5 µm in size were considered to possibly contain lysed cells and thus were excluded from our analysis. Using this method, we detected about a 4-fold reduction in intracellular OMV secretion by the Δ*pagL* strain relative to the wild-type strain (Fig. 5B). Intracellular OMV production was restored by the in *trans* expression of *pagL* from its native promoter. On the contrary, the expression of the inactive PagL variant in the Δ*pagL* strain did not increase intracellular vesiculation to wild-type levels (Fig. 5B). We postulated that the apparent difference in vesiculation could be due to an alteration in the subcellular localization of the strains associated with the disruption of the Δ*pagL* strain. We monitored the colocalization of intracellular bacteria with the vacuolar marker, lysosomal associated membrane protein 1 (Lamp-1) (55). Using this method, we found that the majority of intracellular bacteria (approximately 80%) from both strains did not colocalize with Lamp-1 (see Fig. S6A and B in the supplemental material). It has been shown that *Salmonella* cells often escape the SCV of epithelial cells and replicate in the cytoplasm (56–58). Transmission electron microscopy (TEM) at 4 h postinfection with wild-type and Δ*pagL S*. Typhimurium strains showed that the majority of intracellular bacteria from both strains were detected in the cytoplasm (Fig. 6), suggesting that the bacteria also escape the SCV in J774A.1 macrophages.

## DISCUSSION

Remodeling of the OM is one of the ways in which bacteria can adapt to changing environments (32, 33, 40, 53). Blebbing of OMV provides a rapid mean to dispense proteins that are no longer needed or that can be detrimental under the new condition. Furthermore, OMV can be regarded as a long-distance toxin delivery mechanism (1, 2, 8). Various studies have provided compelling data that suggest the presence of selective machinery responsible for cargo selection in bacteria. In this regard, many bacteria were found to preferentially pack proteins into their OMV (10, 11, 14, 15, 59, 60). Other lines of evidence suggested that the OMV cargo selection is not restricted to proteins but extends to lipids, including phospholipids and lipid A (9, 13, 14). These findings imply that membrane remodeling, in which patches containing the OMV cargo are formed, precedes vesiculation (13, 14, 16, 17). In this work, we investigated the effect of membrane remodeling on OMV biogenesis by the lipid A deacylase PagL in *S*. Typhimurium.

PagL is tightly regulated by the PhoPQ two-component system, which is activated in *S*. Typhimurium inside the vacuole (19). Growing the bacteria under natural PagL-inducing conditions would activate other lipid A-modifying enzymes, making it very difficult to draw conclusions (40, 41). For example, PagC, another PhoPQ-activated protein, was proposed to be involved in OMV formation (44). Moreover, some of these modifications were shown to inhibit PagL activity (39). We therefore recombinantly expressed PagL in *S*. Typhimurium. TEM analysis of cells expressing the active version of the PagL revealed a dramatic increase in the amounts of OMV on the periphery of bacteria. This was only observed when cells were grown on solid media and not in liquid (Fig. 3). Under these conditions, there were no differences between cells expressing PagL, PagL_inactive_, or the vector control. This likely occurred due to the diffusion of the OMV to the medium. This explanation is supported by the fact that the levels of secreted OMV were elevated in the culture supernatants of PagL-expressing cells when cultured in liquid medium (Fig. 2).

Intriguingly, the expression of PagL resulted in the accumulation of deacylated lipid A species exclusively in the OMV (Fig. 4). On the contrary, deacylated lipid A forms were not detectable in cells expressing PagL. These results support our hypothesis that the PagL-mediated changes in the structure of the lipid A contributes to generate membrane curvature and thus OMV formation (Fig. 1). The effect of the degree of acylation on the shape of lipid A is well studied. The number of acyl chains in lipid A affects the angle by which the molecule is tilted to the membrane. Reducing the number of lipid A acyl chains decreases the hydrophobic cross-sectional area of the molecule, shifting its shape from a cone to a cylinder or an inverted cone (35, 61). Therefore, we propose that the accumulation of the deacylated lipid A in the OM destabilizes the membrane and forces it to bend. This model is inspired by the effects of lipid topology on membrane curvature that have been described in eukaryotes (36). Lipids like lysophosphatidylcholine and phosphatidylinositol phosphates mediate the formation of positive membrane curvatures due to their inverted conical shape. Conversely, lipids with small polar head groups relative to their hydrophobic moiety mediate negative membrane curvature (36). In this regard, the phospholipase A2 enzymatic activity was found to generate membrane curvatures in the Golgi complex through the accumulation of the released inverted-cone-shaped lysophospholipids in the membrane (62–64). It is tempting to speculate that accumulation of cone-shaped lipid species with large hydrophobic moiety, such as cardiolipins, in the OM inner leaflet is needed to alleviate the PagL-mediated curvature. Indeed, it has been shown that PhoPQ activation results in the accumulation of cardiolipins in the OM of *S*. Typhimurium. Additionally, the PhoPQ-activated acyl transferase PagP generates triacylated acylphosphatidylglycerol species in the OM (32, 33). These molecules, like cardiolipins, have a small polar head and a large lipid moiety and therefore could also participate in the process. Furthermore, it has recently been proposed that in other bacteria, OMV can be generated through the regulation of a phospholipid transporter at the outer membrane (9). Thus, OMV formation is likely a multifactorial process that needs the orchestrated remodeling of lipid A, phospholipids, and proteins.

The increase in vesiculation observed upon PagL induction cannot be simply ascribed to a generalized membrane destabilization phenomenon. We monitored the expression levels of key players in the σ^E^, Cpx, and Rcs pathways, involved in bacterial envelope stress response (50, 51). Our RT-qPCR analysis showed that none of the previous pathways was activated when PagL or PagL_inactive_ was expressed (see Fig. S4 in the supplemental material).

Previous reports suggested that *S*. Typhimurium produces OMV during its intracellular life. Garcia-del Portillo et al. described the secretion of LPS into the intracellular milieu. It was proposed that *S*. Typhimurium exploits the secreted LPS as signaling molecules to interfere with the host cellular pathways (34). Guidi et al. observed the intracellular secretion of CDT in *S*. Typhimurium OMV using immunofluorescence, whereas LPS colocalized with CDT in vesicular structures (28). In our study, we employed immunofluorescence and confocal microscopy to compare the wild-type *S*. Typhimurium strain and a mutant lacking PagL for the intracellular production of OMV. We employed a lower than usual multiplicity of infection (MOI) to avoid the masking of the phenotype by bacterial lysis or macrophage death. Using LPS-specific monoclonal antibody, we tracked and quantified the OMV produced by intracellular bacteria. In agreement with previous reports, we detected the production of OMV by the *S*. Typhimurium wild-type strain. The strain lacking PagL displayed about 4-fold reduction in the release of intracellular OMV compared to the wild-type strain. In *trans* expression of PagL from its native promoter restored intracellular vesiculation in the mutant, whereas expression of the inactive variant of PagL did not complement the phenotype (Fig. 5A and B). To rule out the possibility that this phenotype was due to a defect in the subcellular localization of the *pagL* mutant strain, we examined the intracellular localization of wild-type and Δ*pagL* strains. We employed Lamp-1 as a marker for SCV. Surprisingly, our microscopy analysis showed that the majority of intracellular *S*. Typhimurium wild-type and Δ*pagL* bacteria were located in the cytoplasm of J774.A macrophages. Deletion of *pagL* did not affect the intracellular localization of *S*. Typhimurium (see Fig. S6A and B in the supplemental material). Our TEM analysis of infected macrophages confirmed the results from our Lamp-1 colocalization assay (Fig. 6). Intracellular *S*. Typhimurium is known to reside in the SCV, albeit it was shown that a subpopulation of bacteria escape the SCV and replicate in the cytosol of epithelial cells (57, 58). Our microscopy analysis shows that a significant cytoplasmic subpopulation of *S*. Typhimurium exists in J774.A macrophages at 4 h postinfection. However, our experiments do not show which subpopulation of bacteria is responsible for intracellular OMV secretion. Although the OMV appear to be distributed throughout the macrophage, OMV might be produced in the cytoplasm or by bacteria within the SCV followed by their release into the cytoplasm upon lysis of the SCV. Our preferred hypothesis is that PagL is induced in the SCV, where it remains latent due to the modifications of lipid A mediated by other PhoPQ-induced proteins. Once bacteria are released from the SCV, LPS starts to be recycled. PagL, which appears to be very stable, might able to deacylate the new lipid A molecules with subsequent OMV biogenesis. Future work will be needed to investigate the spatial and temporal location of OMV production. Other studies will be also needed to understand the relevance of OMV in pathogenesis, which is beyond the scope of this work.

Together, the above results suggest a role for lipid A deacylation in OMV biogenesis. A previous study suggested that the envelope remodeling events that occur during *S*. Typhimurium growth and division might be responsible for OMV release (65). As mentioned previously, OMV might be generated through the regulation of a phospholipid transporter at the OM (9). These models are not mutually exclusive, and it would be interesting to analyze if these mechanisms are synergistically regulating OMV production. Lipid A deacylation can be achieved by induction of lipid A deacylases and also by repression of acyltransferases like LpxM, which has been shown to be silenced upon infection in bacteria such as *Yersinia* (66). PagL orthologs have already been identified in multiple bacteria, suggesting that lipid A remodeling could be involved in OMV biogenesis in diverse bacterial species (42).

## MATERIALS AND METHODS

### Cell culture and media.

J774A.1 mouse macrophages were maintained in maintained in Dulbecco’s modified Eagle medium (DMEM [Life Technologies]) supplemented with 10% fetal bovine serum (FBS) at 37°C in a humid atmosphere of 5% CO_2_. *S. enterica* subsp. *enterica* serovar Typhimurium 14028s was routinely cultured in Luria-Bertani (LB) medium. When needed, antibiotics were supplemented at the following concentrations: ampicillin, 100 µg ml^−1^; and spectinomycin, 100 µg ml^−1^.

### OMV purification.

Cultures from different *S*. Typhimurium strains were normalized by their OD_600_ readings. Next, cells were pelleted down at 5,000 rpm 4 C. In order to remove residual cells, the supernatant was filtered using a 0.45-µm-pore-size polyvinylidene difluoride (PVDF) membrane followed by a 0.2-µm PVDF membrane (Millex GV; Millipore). The filtrate was subjected to ultracentrifugation at 100,000 × *g* for 3 h to harvest OMV (Optima L-90K ultracentrifuge; Beckman Coulter). The supernatant was discarded, the vesicle pellet was washed with sterile PBS, and the ultracentrifugation step was repeated. The final vesicle pellet was resuspended in PBS.

### MALDI-MS analysis of lipid A.

Lipid A from vesicles and cells was prepared in duplicates using 10 mg of sample for each preparation according to the procedure of Yi and Hackett (67). The purified lipid A was resuspended in 6 µl of methanol-dichloromethane (1:1). One microliter of the mixture was loaded on the matrix-assisted laser desorption ionization (MALDI) plate followed by addition of 0.5 µl of 2,4,6-trihydroxyacetophenone monohydrate (THAP) as the matrix. MALDI-mass spectrometry (MS) was then performed on a Bruker Daltonics (Bremen, Germany) UltrafleXtreme MALDI tandem time of flight (TOF/TOF) mass spectrometer in the linear negative mode.

### Cloning of PagL constructs.

PagL was PCR amplified from the genome of *S*. Typhimurium 14028s using primers PagLEcoRIFw (ccccgaattcATGTATATGAAGAGAATATTTATATATC) and PagLBamHIRv (ccccggatccttagtggtggtggtggtggtgGAAATTATAACTAATTGAAGCACC). The reverse primer included extra sequence coding for 6 histidine residues at the C-terminal end of the protein. To include the native promoter of PagL in the construct, primer PagLEcoRIFw was replaced by PagLpromEcoRIFw (aattgaattcACAATGTGACATAACAGAAGTG). The amplified product was restricted with EcoRI and BamHI, followed by cloning in pEXT20 (68) to yield pWel1 (with PagL expressed from the *tac* promoter) and pPagL (with PagL expressed from the native promoter). Four random nucleotides were added at the 5' end before the restriction site in each primer. Restriction sites, the sequence coding for the histidine tag, and the additional four nucleotides at the 5' end are all shown in lowercase letters in the aforementioned primer sequences. To generate a catalytically inactive variant of PagL, site-directed mutagenesis was used. pWel1 was used as the template for primers PagLH163AS165AFw (ACAGAAGCTTATATCCGGGCCTTCGCGAATGGATCACTTACGG) and PagLH163AS165ARv (CCGTAAGTGATCCATTCGCGAAGGCCCGGATATAAGCTTCTGT) to change the histidine and serine residues, at positions 163 and 165, respectively, to alanine. The mutation was confirmed by Sanger sequencing, and the new construct was named pMFH19. Similarly, pPagL was used as a template to generate pPagL_inactive_, which codes for an inactive variant of PagL downstream of its native promoter. For expression in *S*. Typhimurium, *pagL* and *pagL*_inactive_ were subcloned from pWel1 and pMFH19 using the same cloning restriction sites into the low-copy-number vector pEXT21 (68), to yield pWel2 and pWel6, respectively.

### *In vitro* lipid A deacylation assay.

The deacylase activity of different PagL constructs was tested as shown before with some modifications (19). In brief, total membranes were purified from different *E. coli* DH5ɑ strains carrying pWel1, pMFH19, and pEXT20 grown to early stationary phase after cell disruption of the cultures. Protein content of different membrane preparations was determined using 2D-quant kit (GE Health). *S*. Typhimurium 14028s LPS was extracted and used as a substrate for 5 µg of the total membrane proteins obtained from different *E. coli* strains. The reaction mixture was completed to 10 µl with 50 mM HEPES (pH 8.0), 0.1% Triton X-100, 0.5 M NaCl, and 0.5 mM phenylmethylsulfonyl fluoride. All reaction mixtures were incubated at 30°C overnight, followed by lipid A extraction and MS analysis as stated above.

### Immunoblotting.

To test expression of PagL constructs in *S*. Typhimurium, whole-cell pellets were obtained from different strains after normalization based on OD_600_ values. The harvested cell pellets were solubilized by boiling in 1× Laemmli buffer and then loaded on 12% SDS-PAGE gel. Following separation, proteins were transferred to a nitrocellulose membrane, and the PagL constructs were visualized using anti-His polyclonal rabbit antibody (Rockland) followed by IRDye 680-labeled anti-rabbit goat antibody (LI-COR Biosciences, Lincoln, NE). Images were taken using the LI-COR Odyssey Imaging system (LI-COR Biosciences, Lincoln, NE).

### KDO-based quantification of OMV.

To compare the amount of OMV produced by different *S*. Typhimurium strains, OMV were purified essentially as described above with some modifications. Cultures were grown to the early exponential phase, followed by 4 h of induced expression of PagL constructs from pWel2 and pWel6 using 0.5 mM isopropyl β-d-1-thiogalactopyranoside (IPTG). After 4 h, OD_600_ readings were determined. OMV were harvested from different strains after normalizing them according to the OD_600_ readings of the cultures. Final OMV pellets were resuspended in 100 µl of PBS, and OMV production was determined by quantifying the KDO content in different preparations using the method of Lee and Tsai (47). In brief, 50 µl of OMV preparations was boiled for 8 min with 0.5 M H_2_SO_4_ to release the KDO content, followed by oxidation with 50 µl of 0.1 M periodic acid. The latter reaction will convert KDO to formylpyruvic acid. The addition of 0.2 M sodium arsenite and freshly prepared thiobarbituric acid (0.6%), followed by boiling, will yield a chromogen. The reaction product can be extracted using *n*-butanol and measured spectrophotometrically at 552 nm and 509 nm. Commercially available KDO (Sigma-Aldrich) was used as a standard. To measure the KDO concentration in OMV preparations, the 552-nm readings were subtracted by absorbance at 509 nm and the standard curve was used to calculate the KDO content. The KDO concentrations of different OMV preparations were normalized by that of the *S*. Typhimurium strain carrying the empty vector to obtain OMV fold production relative to the control strain. The results were obtained from three independent experiments under the same conditions. A one-way analysis of variance (ANOVA) followed by Tukey’s test to assess the significance of differences between groups was performed. The differences were considered significant when *P* was <0.05.

### RT-mediated qPCR.

RT-mediated qPCR was carried out as described before with some modifications (69). Total RNA was harvested from different strains at mid-log phase. As a positive control for envelop stress response, mid-log-phase *S*. Typhimurium culture was subjected to a cold shock at 4°C for 1 h. cDNA was synthesized using a SuperScript II cDNA synthesis kit according to the manufacturer’s instructions (Invitrogen). Quantitative real-time PCR (RT-qPCR) was used to determine differences in the expression of *rpoE*, *cpxR*, and *rcsC* using the housekeeping gene *rpoD* for normalization. Relative quantification qRT-PCR was done as described previously and analyzed using the threshold cycle (ΔΔ*C_T_*) method (70). For each experiment, two biological replicates were included and the average was presented. The primers used are listed in Table S1 in the supplemental material.

### Construction of Δ*pagL* strain.

An isogenic *S*. Typhimurium mutant lacking *pagL* was created as described before (71). In brief, the FLP recombination target (FRT)-flanked kanamycin resistance (Km^r^) cassette was PCR amplified from pKD4 using primers PagLKOFw (5′-CCATAGGGTCGATAACGATCGGCTATTCACAACACGTTTTGTAGACAACGTACGGTGATTAATTACTCCTTCAGCCAGCAACTCGCTAATTGTTATTCAACTTCAGAACATATGAATATCCTCCTTAGTTCCTATTCCG) and PagLKORv (5′-GTAGTGTGGATGCTATATCAGCCGTTTCTGTGAGCGTAAGCGTGGCGTAGAAAATTTTAAATATGTTAGCCGGTTAAAAATAACTATTGACATTGAAATGGTGGTGGAAGCGATTGTGTAGGCTGGAGCTGCTTCG). The latter primers included around 100 bp (underlined) homologous to the sequences flanking *pagL* in the genome. The amplified product was used to electroporate *S*. Typhimurium 14028s wild-type strain expressing Red recombinase from pKD46. The recombinase-mediated allelic exchange resulted in the replacement of *pagL* by the kanamycin resistance cassette. Km^r^ transformants were selected and cured from pKD46 by growth at 37 C. The kanamycin resistance cassette was excised by the expression of FLP recombinase from pFLP2. The Δ*pagL* strain was cured from pFLP2 by growth on 10% sucrose, which will induce the expression of the toxic SacB. The clean deletion of *pagL* was confirmed by PCR amplification of its flanking regions in the genome followed by sequencing of the amplified product.

### *In vitro* cell culture infection model.

Cell line J774A.1 mouse macrophages (ATCC TIB-67; ATCC, Rockville, MD) were seeded in a 24-well tissue culture plate in Dulbecco’s modified Eagle medium (DMEM) supplemented with 10% fetal bovine serum (FBS) at a density of 5 × 10^5^ cells per well overnight on 13-mm coverslips. The medium was changed to serum-free DMEM, and infection was carried out as shown before with slight modifications (72). Different *S*. Typhimurium strains were cultured overnight in Luria-Bertani (LB) broth at 37°C. The next day, the strains were subcultured until the early log phase. Then, different cultures were washed and resuspended in DMEM to a final concentration of 0.4 OD unit/ml*.* The macrophages were infected at a multiplicity of infection (MOI) of 10:1. The culture plate was centrifuged for 1 min at 1,000 rpm and incubated at 37°C for 30 min. Following incubation, fresh DMEM with high concentration of gentamicin (100 µg/ml) was added for 1 h to kill extracellular bacteria. Next, the medium was changed to lower the gentamicin concentration (50 µg/ml), and incubation was continued for 3 h. After incubation, the cells were fixed with 4% paraformaldehyde PFA) for 20 min, blocked in 2% goat serum and 1% bovine serum albumin (BSA) (15 min). The intracellular salmonellae were stained with primary monoclonal antibody directed against abequose (mouse monoclonal, 1:300 dilution, 0.1% Triton, 0.2% goat serum, 0.1% BSA) for 1 h, followed by an incubation with the secondary antibody (anti-mouse Alexa 488, 1:500 dilution in 0.1% Triton, 0.2% goat serum, and 0.1% BSA). To stain Lamp-1, rabbit polyclonal antibody was used (ab24170; Abcam), followed by incubation with secondary antibody (Alexa Fluor 546-labeled anti-rabbit). Actin was stained with Alexa Fluor 546- or Alexa Fluor 647-labeled-phalloidin (1:40 dilution, 0.1% Triton, 0.2% goat serum, and 0.1% BSA) for 30 min and DAPI (4′,6-diamidino-2-phenylindole [1:1,000 dilution], 0.1% Triton, 0.2% goat serum, 0.1% BSA) was used to stain the nuclei. Slides were examined blindly using an Olympus IX-81 microscope with a Yokagawa spinning disk confocal head, 60× oil immersion lens with a 1.42 numerical aperture, and a Hamamatsu electron multiplying charge-coupled device (EMCCD) camera. Images were taken with equal exposure time without saturation and analyzed with Volocity imaging software. Illustrations were formatted, for noise reduction and increased sharpness, using ImageJ (54). To determine size selection using ImageJ, scale bars were used to set the scale for distance in pixels. The minimal pixel size the Hamamatsu EMCCD camera can detect is 149 nm. Therefore, the imaged vesicles ranged from 149 nm to 250 nm, while any LPS-positive structures ranging from 250 nm up to 0.5 µm were considered cell debris and the whole macrophage was discarded. The images were exported into TIFF format with planes merged, transformed into Grayscale, and split into the 3 channels, with the threshold adjusted until the bacteria were saturated, and vesicles were counted using the predetermined scale. For pictures with multiple macrophages with vesicles, each macrophage was measured separately. The intracellular localization of all the counted subjects was confirmed by confocal microscopy examination using z-stacks. A total of 30 images were used to assess intracellular vesicle production by each strain. To validate our method, fluorescent beads (Polysciences, Warrington, PA) of 1-µm diameter were imaged under the same conditions. The size of the beads was confirmed by using the scale bar measurement set from the *Salmonella* vesicle images.

### Transmission electron microscopy.

For negative staining and analysis by transmission electron microscopy, bacterial suspensions were allowed to absorb onto freshly glow-discharged Formvar/carbon-coated copper grids for 10 min. Grids were washed in dH_2_O and stained with 1% aqueous uranyl acetate (Ted Pella, Inc., Redding, CA) for 1 min. Excess liquid was gently wicked off, and grids were allowed to air dry. Samples were viewed on a JEOL 1200EX transmission electron microscope (JEOL United States, Peabody, MA) equipped with an AMT 8-megapixel digital camera (Advanced Microscopy Techniques, Woburn, MA). To examine *Salmonella*-infected host cells, J774A.1 mouse macrophages were infected with *S*. Typhimurium as mentioned above. At 4 h postinfection, infected cells were fixed overnight at 4°C in 4% glutaraldehyde–2% paraformaldehyde, 0.2 M sucrose, and 4 mM CaCl_2_ in 0.16 M sodium cacodylate buffer (pH 7.4). Following washing with 0.05 M sodium cacodylate, lipids were then fixed with 1% ice-cold osmium tetroxide (OsO_4_) in 0.05 M sodium cacodylate buffer. Coverslips were blocked in en bloc stain with 1% uranyl acetate in 0.1 M sodium acetate buffer (pH 5.2) for 15 min Sodium acetate (0.1 M) was used to wash the coverslips, followed by Milli-Q filtered water and increasing ethanol concentrations in propylene oxide. Cells were permeated with a mixture of EMbed 812 and Araldite 502 resins and embedded on gelatin capsules. Thermal polymerization was performed at 60°C for 48 h. Ultrathin sections with a thickness of 60 nm were generated using a Leica UC7 ultramicrotome (Leica Microsystems, Inc.) and contrasted with 2% uranyl acetate and Reynolds’ lead citrate. Sections were imaged using a Hitachi H-7650 transmission electron microscope (Hitachi-High Technologies) at 80 kV and a 16-megapixel TEM camera (XR111; Advanced Microscopy Techniques, Woburn, MA).

## SUPPLEMENTAL MATERIAL

Figure S1  *S*. Typhimurium OMV lacks deacylated lipid A forms under standard laboratory conditions. Lipid A was extracted from cells and OMV of the *S*. Typhimurium wild-type strain, followed by MALDI-MS analysis. Lipid A from both fractions displayed the *bis-*phosphorylated hexa-acylated lipid A form (*m*/*z* 1,796) as the most abundant form. Other known lipid A derivatives were detected, including *bis-*phosphorylated hepta-acylated lipid A (*m*/*z* 2,035) and monophosphorylated hexa-acylated lipid A (*m*/*z* 1,716). None of the known deacylated lipid A species was detected in both fractions. Download Figure S1, TIF file, 2.4 MB

Figure S2  Loss of the deacylase activity in PagL_Inactive_. Total membranes were purified from different *E. coli* DH5ɑ strains carrying pWel1 (A), pMFH19 (B), and pEXT20 (C). *S*. Typhimurium LPS was used as a substrate to test the deacylase activity of different membrane preparations. One reaction mixture contained only *S*. Typhimurium LPS without the addition of *E. coli* membranes as a control (D). Following overnight incubation, lipid A was purified from different reaction mixtures and analyzed by MS. Download Figure S2, TIF file, 2.5 MB

Figure S3  PagL and its inactive variant are expressed at equal levels in *S*. Typhimurium. Cell lysates were obtained from *S*. Typhimurium strains carrying pWel2 (expressing PagL), pWel6 (expressing PagL_inactive_), and empty vector (VC), followed by SDS-PAGE separation. The expression levels of PagL and PagL_inactive_ in *S*. Typhimurium were monitored by immunoblotting using anti-His rabbit antibody (primary antibody) followed by IRDye 680-labeled anti-rabbit goat antibody (secondary antibody). As shown, both proteins were expressed at similar levels. Download Figure S3, TIF file, 1.2 MB

Figure S4  PagL expression does not activate σ^E^, Cpx, or Rcs pathways in *S*. Typhimurium. Total RNA was purified from different *S*. Typhimurium strains at mid-log phase using a phenol extraction method. Following cDNA synthesis, relative expression of different genes was determined by qPCR using SYBR green and the ΔΔ*C_T_* method. *rpoD* was employed as an endogenous control. Expression of PagL and PagL_inactive_ was induced by IPTG from pWel2 and pWel6, respectively, for 4 h. “VC” denotes the vector control, pEXT21. Two biological replicates were employed for each strain, and the mean ± standard deviation (SD) is presented. As a positive control, cDNA obtained from the *S*. Typhimurium wild-type strain after a 60-min cold shock was used as a template. Download Figure S4, TIF file, 2.4 MB

Figure S5  OMV of *S*. Typhimurium harboring pEXT21 share a similar lipid A content to that of cells. Lipid A was extracted from cells and OMV of *S*. Typhimurium carrying pEXT21, followed by MALDI-MS analysis. *bis*-Phosphorylated hexa-acylated lipid A (*m*/*z* 1,796) was the most abundant form in both compartments. Download Figure S5, TIF file, 2 MB

Figure S6  The wild-type (WT) and Δ*pagL S*. Typhimurium strains were detected mainly in the cytoplasmic compartment of J774A.1 mouse macrophages. At 4 h postinfection, infected macrophages were fixed with 4% PFA. Lamp-1 (red) was used as a marker for SCV, while LPS was stained to visualize bacteria (green) with DAPI (blue) used to stain the nuclei. Actin (gray) was stained by Alexa Fluor 647-labeled phalloidin to visualize the cell boundaries, and thus only intracellular bacteria were used in the analysis. Scale bars represent 5 µm (A). A total of 50 images per strain were used to quantify the Lamp-1-colocalized bacteria relative to the total intracellular population (B). Presented are means ± SEM. Download Figure S6, TIF file, 2.8 MB

Table S1  Primers used in the RT-qPCR analysis of different genes involved in the bacterial envelope stress response. Table S1, DOCX file, 0.01 MB
