# Supplementary figures and images for: LPS Remodeling Triggers Formation of Outer Membrane Vesicles in Salmonella
Source: mBio. 2016 Jul 12;7(4):e00940-16. doi: 10.1128/mBio.00940-16 (PMC4958258; doi:10.1128/mBio.00940-16)

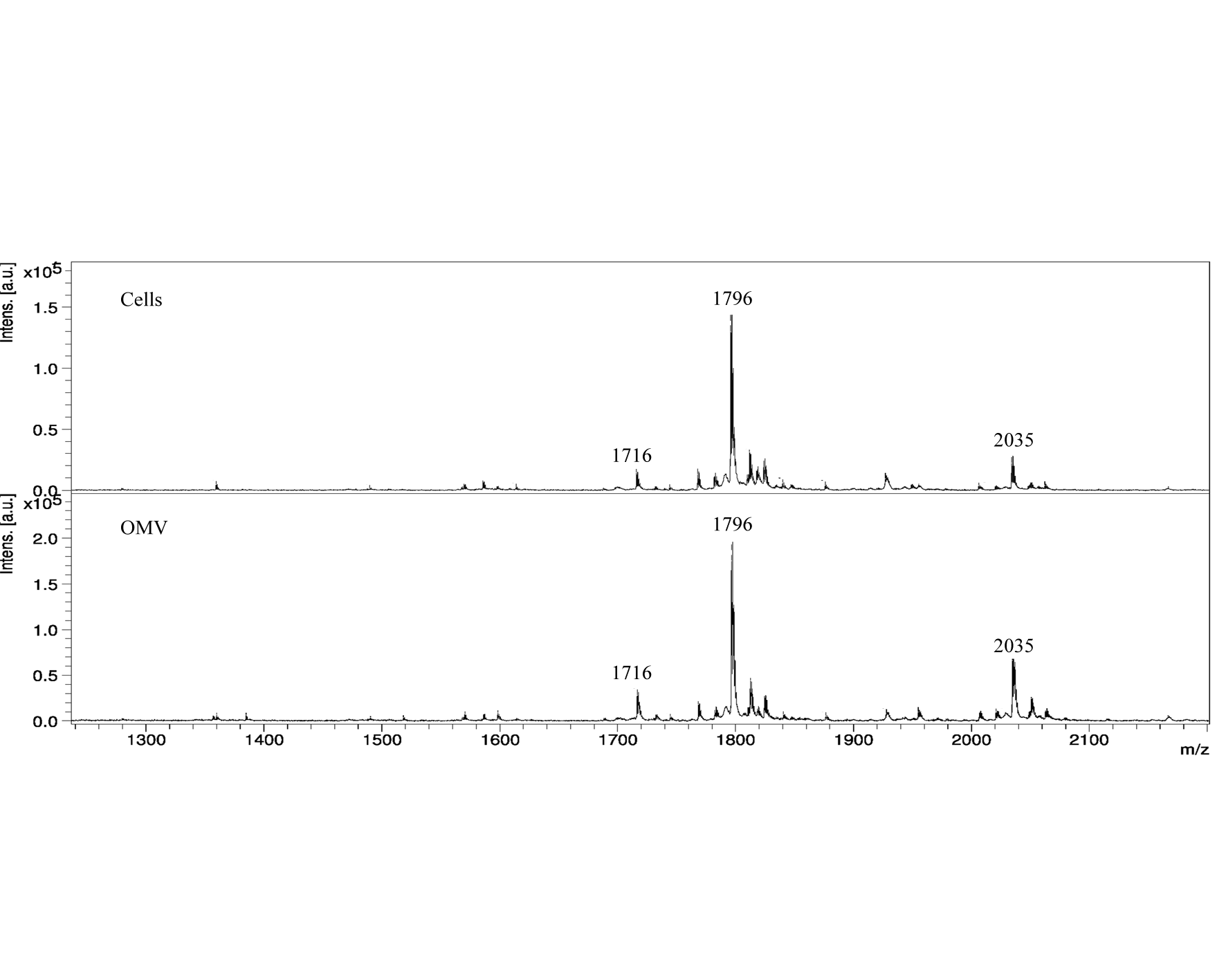

Supplement: Figure S1 — S. Typhimurium OMV lacks deacylated lipid A forms under standard laboratory conditions. Lipid A was extracted from cells and OMV of the S. Typhimurium wild-type strain, followed by MALDI-MS analysis. Lipid A from both fractions displayed the bis-phosphorylated hexa-acylated lipid A form (m/z 1,796) as the most abundant form. Other known lipid A derivatives were detected, including bis-phosphorylated hepta-acylated lipid A (m/z 2,035) and monophosphorylated hexa-acylated lipid A (m/z 1,716). None of the known deacylated lipid A species was detected in both fractions. Download [file mbo003162890sf1.tif]

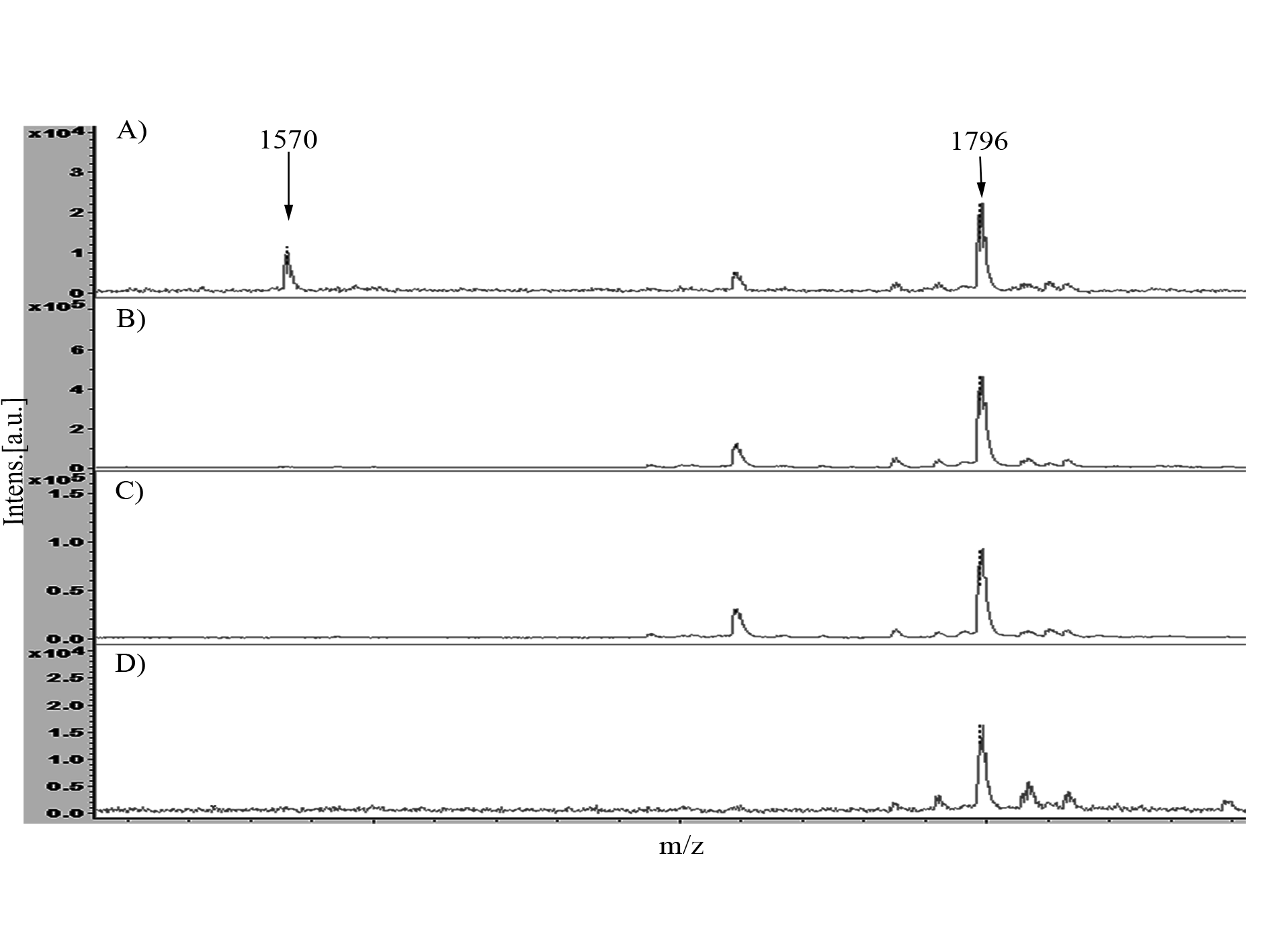

Supplement: Figure S2 — Loss of the deacylase activity in PagLInactive. Total membranes were purified from different E. coli DH5ɑ strains carrying pWel1 (A), pMFH19 (B), and pEXT20 (C). S. Typhimurium LPS was used as a substrate to test the deacylase activity of different membrane preparations. One reaction mixture contained only S. Typhimurium LPS without the addition of E. coli membranes as a control (D). Following overnight incubation, lipid A was purified from different reaction mixtures and analyzed by MS. Download [file mbo003162890sf2.tif]

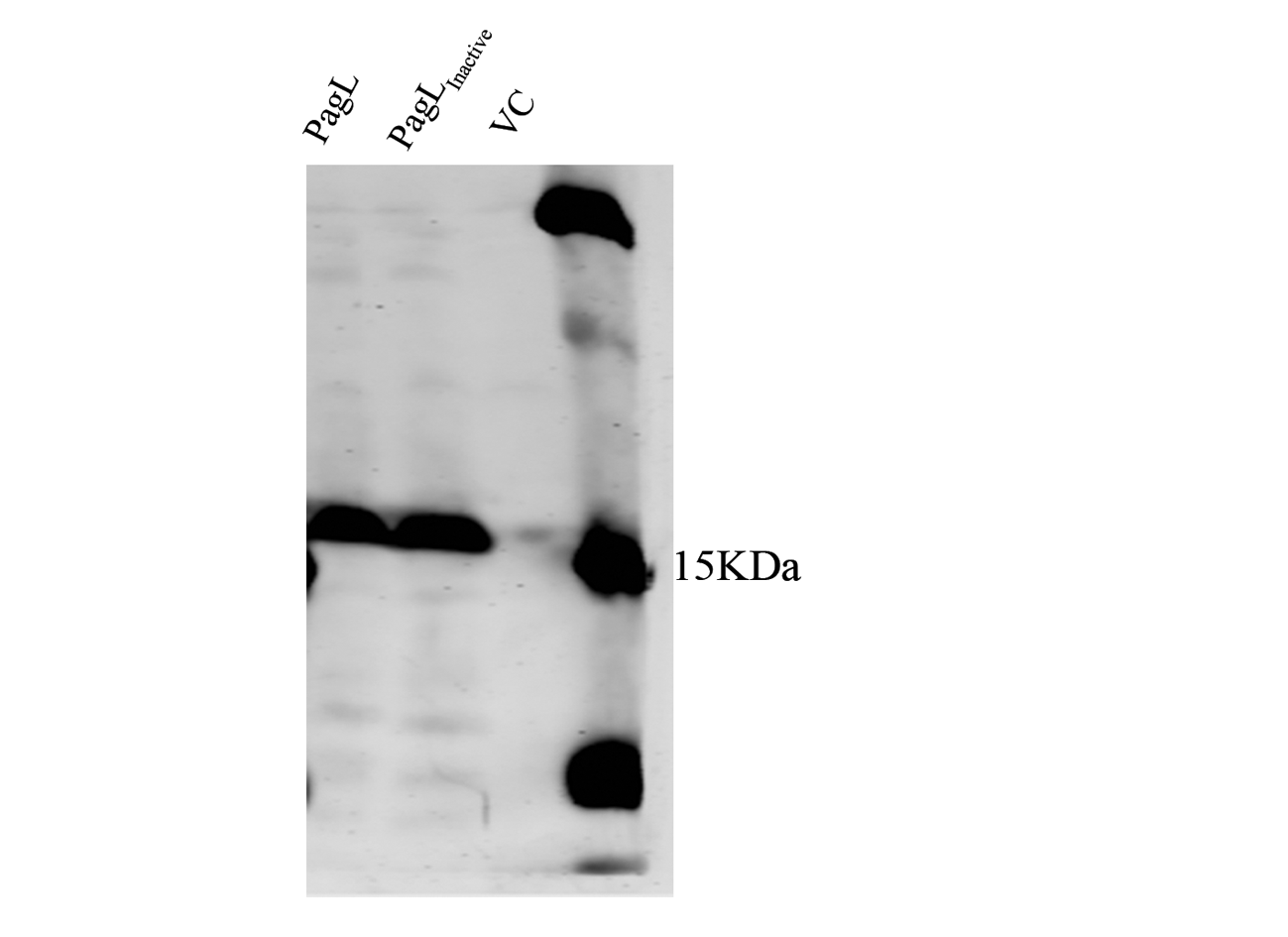

Supplement: Figure S3 — PagL and its inactive variant are expressed at equal levels in S. Typhimurium. Cell lysates were obtained from S. Typhimurium strains carrying pWel2 (expressing PagL), pWel6 (expressing PagLinactive), and empty vector (VC), followed by SDS-PAGE separation. The expression levels of PagL and PagLinactive in S. Typhimurium were monitored by immunoblotting using anti-His rabbit antibody (primary antibody) followed by IRDye 680-labeled anti-rabbit goat antibody (secondary antibody). As shown, both proteins were expressed at similar levels. Download [file mbo003162890sf3.tif]

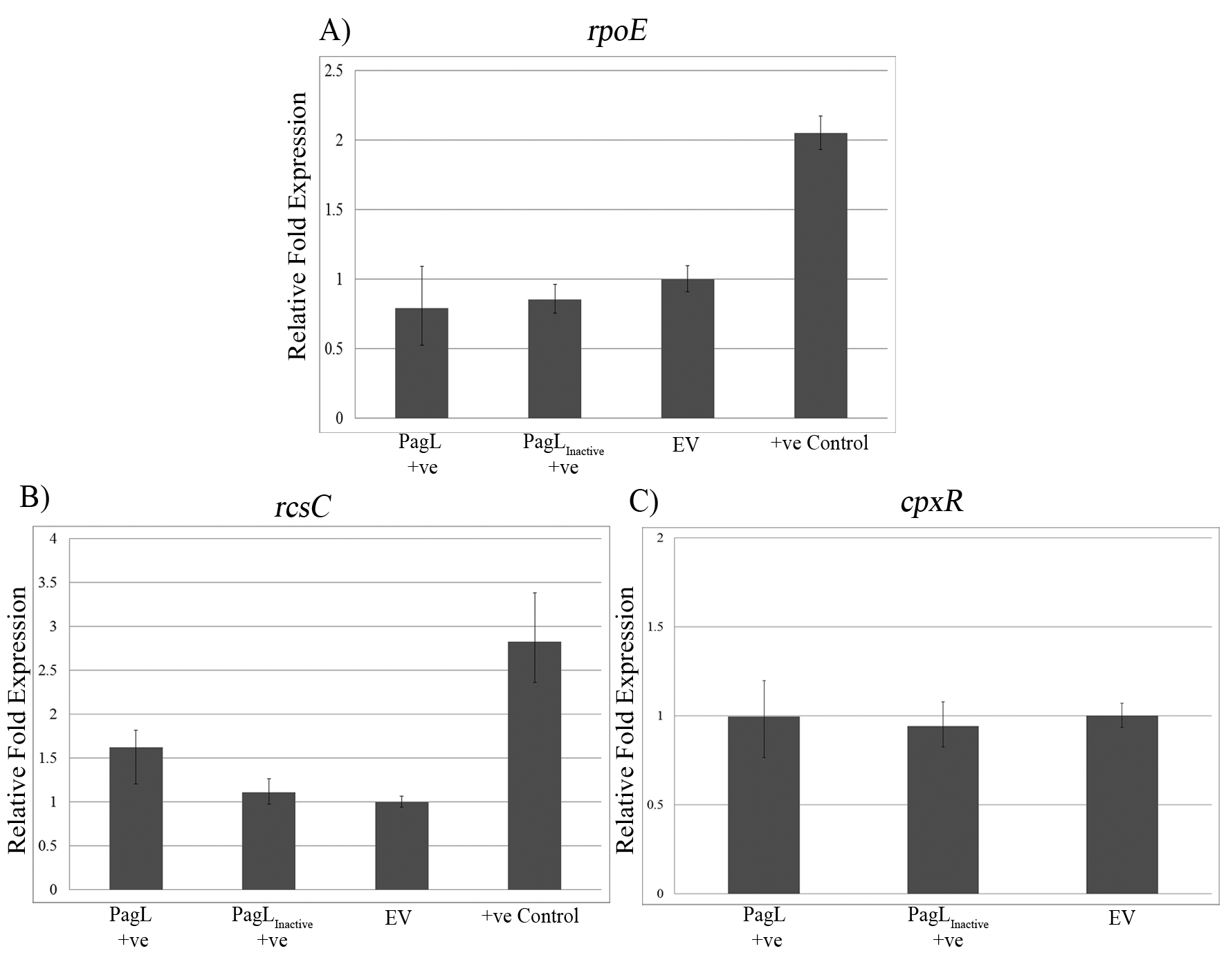

Supplement: Figure S4 — PagL expression does not activate σE, Cpx, or Rcs pathways in S. Typhimurium. Total RNA was purified from different S. Typhimurium strains at mid-log phase using a phenol extraction method. Following cDNA synthesis, relative expression of different genes was determined by qPCR using SYBR green and the ΔΔCT method. rpoD was employed as an endogenous control. Expression of PagL and PagLinactive was induced by IPTG from pWel2 and pWel6, respectively, for 4 h. “VC” denotes the vector control, pEXT21. Two biological replicates were employed for each strain, and the mean ± standard deviation (SD) is presented. As a positive control, cDNA obtained from the S. Typhimurium wild-type strain after a 60-min cold shock was used as a template. Download [file mbo003162890sf4.tif]

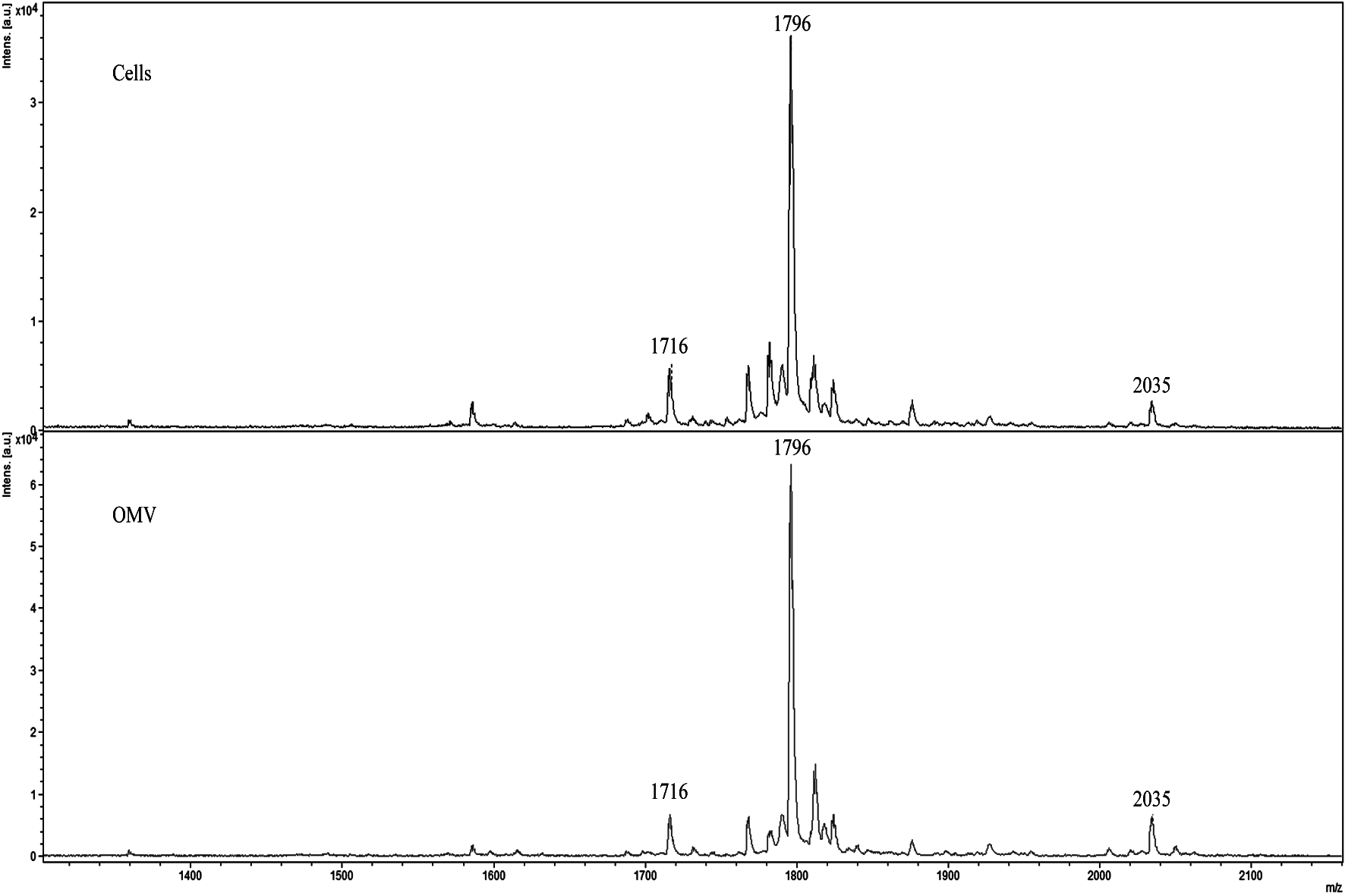

Supplement: Figure S5 — OMV of S. Typhimurium harboring pEXT21 share a similar lipid A content to that of cells. Lipid A was extracted from cells and OMV of S. Typhimurium carrying pEXT21, followed by MALDI-MS analysis. bis-Phosphorylated hexa-acylated lipid A (m/z 1,796) was the most abundant form in both compartments. Download [file mbo003162890sf5.tif]

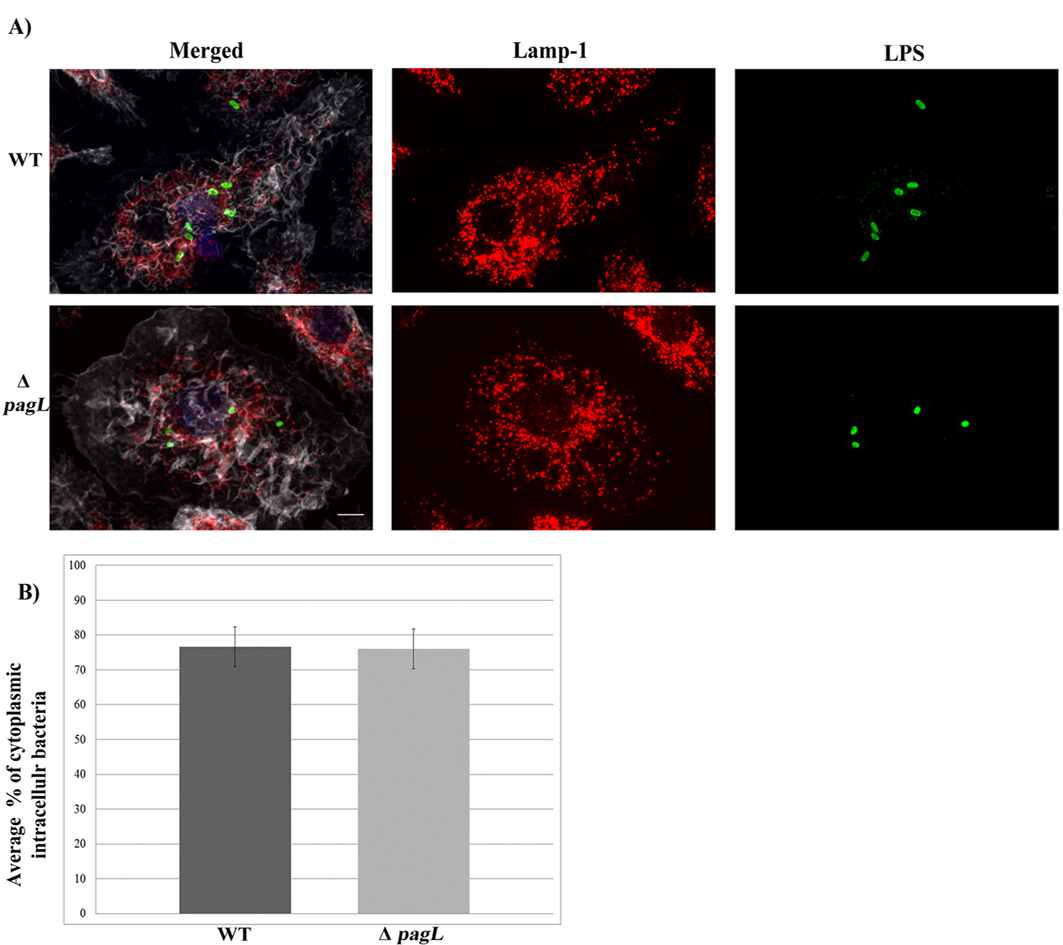

Supplement: Figure S6 — The wild-type (WT) and ΔpagL S. Typhimurium strains were detected mainly in the cytoplasmic compartment of J774A.1 mouse macrophages. At 4 h postinfection, infected macrophages were fixed with 4% PFA. Lamp-1 (red) was used as a marker for SCV, while LPS was stained to visualize bacteria (green) with DAPI (blue) used to stain the nuclei. Actin (gray) was stained by Alexa Fluor 647-labeled phalloidin to visualize the cell boundaries, and thus only intracellular bacteria were used in the analysis. Scale bars represent 5 µm (A). A total of 50 images per strain were used to quantify the Lamp-1-colocalized bacteria relative to the total intracellular population (B). Presented are means ± SEM. Download [file mbo003162890sf6.tif]
