# Supplementary material for: LPS Remodeling Triggers Formation of Outer Membrane Vesicles in Salmonella
Source: mBio. 2016 Jul 12;7(4):e00940-16. doi: 10.1128/mBio.00940-16 (PMC4958258; doi:10.1128/mBio.00940-16)
Supplement: Table S1 — Primers used in the RT-qPCR analysis of different genes involved in the bacterial envelope stress response. [file mbo003162890st1.docx]

**Table S1. Primers used in the RT-qPCR analysis of different genes involved in the bacterial envelope stress response.**

| rpoEfw | GAAAGAAATTTCGAACCCTGAGAA |
| --- | --- |
| rpoErv | TCCCGTAAGGTGATTGCCATA |
| cpxRfw | GGGCGCGGACGACTATT |
| cpxRrv | AGTGGGAACGGCGCAAA |
| rcsCfw | TCGCGAACTGGTACTCAAAAAA |
| rcsCrv | CCAGGACTAAATCTACAGGCACAGA |
| rpoDfw | GCGGCTACAAATTCTCCACCTA |
| rpoDrv | CAATCATATGCACCGGAATACG |
